# Reactive Oxygen Species and Autophagy Associated Apoptosis and Limitation of Clonogenic Survival Induced by Zoledronic Acid in Salivary Adenoid Cystic Carcinoma Cell Line SACC-83

**DOI:** 10.1371/journal.pone.0101207

**Published:** 2014-06-25

**Authors:** Xi-Yuan Ge, Lin-Qian Yang, Yang Jiang, Wen-Wen Yang, Jia Fu, Sheng-Lin Li

**Affiliations:** 1 Central Laboratory, Peking University School and Hospital of Stomatology, Haidian District, Beijing, PR China; 2 Department of Oral and Maxillofacial Surgery, Peking University School and Hospital of Stomatology, Haidian District, Beijing, PR China; Central South University, China

## Abstract

Salivary adenoid cystic carcinoma is an epithelial tumor in the head and neck region. Despite its slow growth, patients with salivary adenoid cystic carcinoma exhibit poor long term survival because of a high rate of distant metastasis. Lung and bone are common distant metastasis sites. Zoledronic acid, a third generation bisphosphonate, has been used for tumor-induced osteolysis due to bone metastasis and has direct antitumor activity in several human neoplasms. Here, we observed that zoledronic acid inhibited salivary adenoid cystic carcinoma cell line SACC-83 xenograft tumor growth in nude mice. In vitro, zoledronic acid induced apoptosis and reduced clonogenic survival in SACC-83. Flow cytometry and western blotting indicated that the cell cycle was arrested at G0/G1. Zoledronic acid treatment upregulated reactive oxygen species as well as the autophagy marker protein LC-3B. Reactive oxygen species scavenger N-acetylcysteine and autophagy antagonist 3-methyladenine decreased zoledronic acid-induced apoptosis and increased clonogenic survival. Silencing of the autophagy related gene Beclin-1 also decreased zoledronic acid-induced apoptosis and inhibition of clonogenic formation. In addition, isobolographic analysis revealed synergistic effects on apoptosis when zoledronic acid and paclitaxel/cisplatin were combined. Taken together, our results suggest that zoledronic acid induced apoptosis and reduced clonogenic survival via upregulation of reactive oxygen species and autophagy in the SACC-83 cell line. Thus, zoledronic acid should be considered a promising drug for the treatment of salivary adenoid cystic carcinoma.

## Introduction

Salivary adenoid cystic carcinoma (SACC) is a malignant tumor that arises from the secretory epithelial cells of salivary glands. It accounts for less than 1% of all head and neck malignancies and approximately 10–20% of all salivary neoplasms [1]–[3]. The occurrence of adenoid cystic carcinoma in other secretory glands (e.g. breast, colon, prostate) is very rare [3]. SACC is characterized by slow local growth, a high incidence of perineural invasion, infrequent regional metastases, frequent local recurrence and mostly slowly progressive, relatively indolent distant metastasis [4]. About 40–60% of SACC patients develop distant metastases [5]. Late distant metastases are the primary cause of the rather low long term survival rate [6]. Lung is the most common distant metastasis site, followed by bone and other sites including liver, brain, thyroid, spleen and pancreatic gland [5]–[7]. SACC is currently incurable and most patients will eventually succumb to local recurrence, distant metastases or both [7]. Unlike lung metastasis, the course of the disease is usually fulminant if metastases occur in bone, especially in the spine [7], [8].

Bisphosphonates are currently the most important class of inhibitors of osteoclast mediated bone resorption [9], [10] and are used extensively for the treatment of skeletal diseases such as Paget’s disease, [10], [11] postmenopausal osteoporosis [10], [12] and tumor-induced osteolysis [10], [13]. Bisphosphonates are pyrophosphate analogs that bind to hydroxyapatite, accumulate in bone and inhibit osteoclastic activity [14]. Zoledronic acid (ZOL) is a third generation nitrogen-containing bisphosphonate. Its core bisphosphonate moiety attaches to bone and its imidazole ring containing two nitrogen atoms confers its potency [14]. Previous studies have shown that ZOL has antitumor activity in several human neoplasms, including myeloma and breast, prostate, colon and pancreatic cancer [10], [15]–[19]. The clinical benefits of ZOL have been extended to patients with bone metastases secondary to a broad range of solid tumors including prostate cancer, lung cancer and renal cell carcinoma [20]. ZOL is the current clinical standard for the prevention of bone metastasis from human cancers [19].

These previous findings suggest that chemotherapy with ZOL might be effective for the treatment or prevention of SACC patients with bone metastasis. However, the effect of ZOL on SACC has not yet been reported. Therefore, the aim of the present study was to investigate the effect of ZOL on an SACC cell line, SACC-83, and the underlying mechanism. In addition, ZOL was combined with paclitaxel/cisplatin to determine whether ZOL has a synergic antineoplastic effect with these two traditional chemotherapeutics in vitro.

## Materials and Methods

### Cell line and reagents

The SACC-83 cell line, which originated from a patient’s sublingual gland SACC cells, was established in 1983 [21]. We further confirmed that the cell line is authentic adenoid cystic carcinoma cell line by short tandem-repeat analysis (STR) and immunostaining [22], [23]. Cells were cultured in RPMI 1640 medium (Gibco, Billings, MT) supplemented with 12% fetal bovine serum (Yuanhengjinma, Beijing, China), 100 U/ml penicillin and 100 µg/ml streptomycin and incubated at 37°C in a humidified atmosphere of 5% CO_2_ in air. ZOL (Sigma–Aldrich, St. Louis, MO) was dissolved in phosphate buffered saline (PBS) and stored as a 10 mM stock solution at –20°C.

### Cell counting

SACC-83 cells were seeded in 96-well plates at an initial density of 2×10^3^ cells/well in 100 µl of culture medium. After treatment, adherent cells were harvested with 0.25% trypsin-EDTA (Gibco) and collected by centrifugation. Non-adherent cells were collected from spent media by centrifugation. Cell pellets were resuspended in PBS, and trypan blue solution (Sigma–Aldrich) was added to a final concentration of 0.04%. Live cells with intact cell membranes were not colored and thus not counted.

### Cell viability assay

SACC-83 cells were seeded in 96-well plates at 2×10^3^ cells/well in 100 µl of culture medium. After 24 h of incubation, the cells were treated in the presence or absence of the different reagents at the concentrations and for the periods indicated. The medium was then removed and the cells were washed twice with fresh medium. Next, 100 µl of fresh serum-free RPMI 1640 medium containing 10 µl of Cell Counting Kit-8 (CCK-8) reagent (Dojindo, Kumamoto, Japan) was added to each well. After 2 h of incubation at 37°C, the optical density (OD) was recorded on an ELx808 Absorbance Microplate Reader (BioTek, Winooski, VT) at 450 nm. Untreated cells were defined as the negative control, and wells containing the CCK-8 reagent and no cells were used as the blank control. The percentage viability of the control was calculated as (OD of treated cells – OD of blank control)/(OD of negative control – OD of blank control)×100.

### Colony formation assay

Cells were plated in 100 mm dishes in triplicate, in their normal medium at an initial density of 300 cells/dish. After treatment with different concentrations of ZOL (0.01 µM, 0.1 µM, 1 µM, 5 µM or 10 µM) for 14 days, the cells were fixed with 70% ethanol for 15 min at room temperature and stained with 4 mg/ml crystal violet (Sigma–Aldrich). The number of colonies was determined from three independent experiments.

### In vivo antitumor effect

The BALB/c female nude mice, 6–8 weeks old and weighing 20–22 g were obtained from Vital River Laboratories (Beijing, China). The animal experiments were approved by the Peking University Institutional Animal Care and Use Committee (Permit Number: LA2014179) and were performed according to the guideline on animal experiments. SACC-83 cells were subcutaneously inoculated (5×10^6^ cells/mouse) in the right flanks of 10 mice. Seven days later, the tumors had growth to 100–250 mm^3^, and the mice were randomized into 3 groups (5 mice/group). The mice were injected subcutaneously with ZOL (50 µg/kg or 200 µg/kg) or the same amount of PBS as control for three times a week for 3 consecutive weeks. Tumor lengths and widths were measured with calipers every three days. The tumor volume was determined by using the formula V (mm^3^) = length×(width)^2^/2.

### Cell cycle analysis

Cells were treated with different doses of ZOL for 24 h, harvested using 0.25% trypsin-EDTA (Gibco), centrifuged at 300 *g* and washed twice with cold PBS. The pellet was resuspended in ice-cold 70% ethanol for at least 12 h. Following two more washes with PBS, samples were incubated with a fluorescent probe solution containing PBS, 0.2% Triton X-100, 50 µg/ml propidium iodide (PI) and 0.1 mg/ml RNase A for 30 min at room temperature in the dark. The cells were then analyzed using an EPICS-XL flow cytometer with excitation at 488 nm and Multicycle software (Beckman Coulter, Brea, CA).

### Western blot analysis

Cells were seeded in 100 mm dishes at 2×10^6^ cells/dish, incubated for 24 h and then treated with different doses of reagent for the indicated times. Cells were harvested by scraping into ice-cold RIPA buffer containing a protease inhibitor cocktail (Applygen, Beijing, China). The protein concentration was measured with a BCA protein assay kit (Applygen) following the manufacturer’s instructions. Sodium dodecyl sulfate polyacrylamide gel electrophoresis (SDS-PAGE) Sample/Loading Buffer 5X was added to the protein samples, which were then heated at 95°C for 5 min. The samples (30 µg) were subjected to SDS-PAGE and transferred to polyvinylidene fluoride membranes. The membranes were blocked in 5% non-fat milk mixed with TBST and washed in TBST before overnight incubation at 4°C with primary antibodies diluted according to the manufacturer’s instructions. Then, the membranes were washed with TBST and incubated with the appropriate secondary antibody (1∶10,000) for 1 h at room temperature. All antibodies were purchased from Epitomics (Burlingame, CA) with the exception of LC-3B and PARP, which were purchased from Beyotime (Haimen, China). After washing with TBST, the membranes were incubated with Supersignal West Pico Chemiluminescent Substrate (Thermo Scientific, Waltham, MA) and images were acquired using a Fusion FX5 system (Vilber Lourmat, Marne-la-Vallée, France). Image-Pro Plus software (Medica Cybernetics Inc., Bethesda, MD, USA) was used to analyze the densitometry data.

### Annexin-V/PI assay

Cell apoptosis was evaluated using an Annexin-V-FLUOS staining kit (Roche, Basel, Switzerland) according to the manufacturer’s instructions. In brief, after treatment with different doses of reagent for 48 h, cells were harvested with 0.5% trypsin; 10^6^ cells were then washed with cold PBS and centrifuged at 200 *g* for 5 min. Twenty microliters of Annexin-V-FLUOS labeling reagent and 20 µl PI solution were prediluted in 1 ml incubation buffer. The cell pellet was resuspended in 100 µl of the solution and incubated for 10 min at room temperature. Then, cell suspensions were analyzed using an EPICS-XL flow cytometer (Beckman Coulter).

### 4,6-Diamidino-2-phenylindole dihydrochloride (DAPI) staining

Cells grown on sterile glass coverslips were treated with different concentrations of reagent for 48 h. Then, the cells were fixed with 10% formaldehyde in PBS for 5 min and incubated with 5 µg/ml DAPI (Sigma–Aldrich) in the dark for 5 min at room temperature. The cells were then gently washed twice with PBS. Nuclear condensation and fragmentation were identified as apoptotic cells and counted within randomly selected fields under a BX51 fluorescence microscope (Olympus, Tokyo, Japan). A minimum of 200 cells were counted and the percentage of apoptotic cells was calculated; the data were presented as the mean ± standard deviation of at least three independent experiments.

### Terminal transferase deoxyuridine triphosphate (dUTP) nick end labeling (TUNEL) assay

Apoptotic cells were examined by fluorescence staining using a TUNEL apoptosis detection kit (Beyotime) following the manufacturer’s instructions. Briefly, after treatment for 48 h, cells cultured on sterile glass coverslips were fixed with 4% paraformaldehyde in PBS for 30 min and then permeabilized with 0.1% Triton X-100 for 2 min at room temperature. Cells were incubated with a reaction mix containing fluorescein isothiocyanate-dUTP and terminal deoxynucleotidyl transferase for 60 min in the dark. Analysis was performed using an LSM 5 Exciter confocal laser scanning microscope (Zeiss, Oberkochen, Germany).

### RNA interference

Chemically synthesized small interfering RNA (siRNA) duplexes specific for human Beclin-1 and Atg 7 were obtained from Guangzhou RiboBio (Guangzhou, China). The sequence of the Atg 7 targeted siRNA was 5′-AGAACGAGAGCGUACUCAA-3′; the sequence of the Beclin-1 targeted siRNA was 5′-GTTCATTTCCAATCCGCCC-3′. The non-targeting control was a siRNA lacking specificity for any known gene target (Guangzhou RiboBio). To achieve gene silencing, we used the transfection reagent Lipofectamine RNAiMAX (Invitrogen, Carlsbad, CA) following the manufacturer’s instructions. Cells were transfected with the siRNA for 24 h, followed by drug treatment. The gene silencing effects were evaluated by western blot analysis.

### Reactive oxygen species (ROS) detection

Levels of intracellular ROS were measured using a Reactive Oxygen Species Assay Kit (Beyotime). Dichlorodihydrofluorescein diacetate (DCFH-DA) is oxidized by ROS in viable cells in which DCF which is highly fluorescent at 530 nm. Briefly, the SACC-83 cells were trypsinized, washed with PBS and incubated with DCFH-DA at a final concentration of 10 µM in RPMI 1640 medium for 30 min at 37°C. Then, the cells were washed three times with PBS and the fluorescence was measured using an EPICS-XL flow cytometer (Beckman Coulter) with an excitation wavelength of 488 nm and an emission wavelength of 530 nm.

### Monodansyl cadaverine (MDC) staining

MDC (Sigma–Aldrich) was used as a probe for the detection of autophagic vacuoles in cultured cells. SACC-83 cells were plated on 35 mm plastic Petri dishes with glass bottoms (Nest, Wuxi, China). After treatment, the medium was replaced by 1 ml of 50 µM MDC in RPMI 1640 medium and the cells were incubated for 10 min at 37°C in the dark. After incubation, the cells were washed twice with PBS and visualized and photographed in the DAPI channel of an LSM 5 Exciter confocal laser scanning microscope (Zeiss).

### LC-3B immunofluorescence staining

SACC-83 cells were seeded onto 35 mm plastic Petri dishes with glass bottoms (Nest). After treatment, the cells were fixed with cold 4% paraformaldehyde for 10 min and blocked with 1% bovine serum albumin dissolved in 0.1% Triton X-100 for 1 h. The cells were incubated with rabbit LC-3B primary antibody (Beyotime) at 4°C overnight and then incubated for 1 h with Alexa Fluor 488-labeled goat anti-rabbit secondary antibody (Beyotime). Samples were viewed and photographed with an LSM 5 Exciter confocal laser scanning microscope (Zeiss).

### Isobolographic analysis

Synergism between ZOL and paclitaxel/cisplatin was evaluated by isobolographic analysis[24]. This method is referred to as a ‘gold standard’ because it is the only model of analysis proven valid for analyzing interactions between biologically active agents [24]–[28]. Briefly, doses of ZOL were plotted on the *x*-axis and doses of paclitaxel/cisplatin were plotted on the *y*-axis, and points representing equal effects on cell viability were connected to obtain an isobologram. A straight line was obtained by connecting the 50% inhibitory concentration (IC50) of ZOL plotted on the *x*-axis (Z1) with that of paclitaxel/cisplatin plotted on the *y*-axis (Z2); all points on this line represent dose pairs that are additive, whereas points below or above the line represent synergism or antagonism, respectively.

In this study, IC50s for SACC-83 were measured by CCK-8 assay. ZOL and paclitaxel/cisplatin were mixed in fixed ratios of IC50 (1∶1, 1∶3 and 3∶1) based on their final concentration. The mixture was then administered in various doses and the response (inhibition of cell viability) was measured to obtain a dose–response relationship for the mixture. Then, the IC50 of the mixture (Zmix) and the theoretical additive IC50 (Zadd) were calculated and the resulting data points plotted on an isobologram. We also compared the relative median potencies of the mixtures and the theoretical additive combinations using SPSS Version 19.0 software (IBM, Armonk, NY). If the difference was statistically significant (i.e. the 95% confidence intervals did not overlap), we concluded that the mixture’s effect departed from simple additivity. This potency of the mixture was then compared with that of the theoretical additive dose, Zadd. If Zmix < Zadd, the mixture was considered to be synergistic at this ratio, whereas Zmix > Zadd represented antagonism. No significant difference meant that the mixture’s effect was simply additive.

### Statistical analysis

All statistical analyses were performed using SPSS Version 19.0 (IBM). All numerical data were expressed as the mean ± standard deviation. One-way analysis of variance was used for the statistical analysis of data, followed by the Bonferroni method for multiple comparisons between indicated pairs. *P* values less than 0.05 were considered statistically significant.

## Results

### ZOL inhibits the viability and colony formation of SACC-83 cells

**Figure 1:**
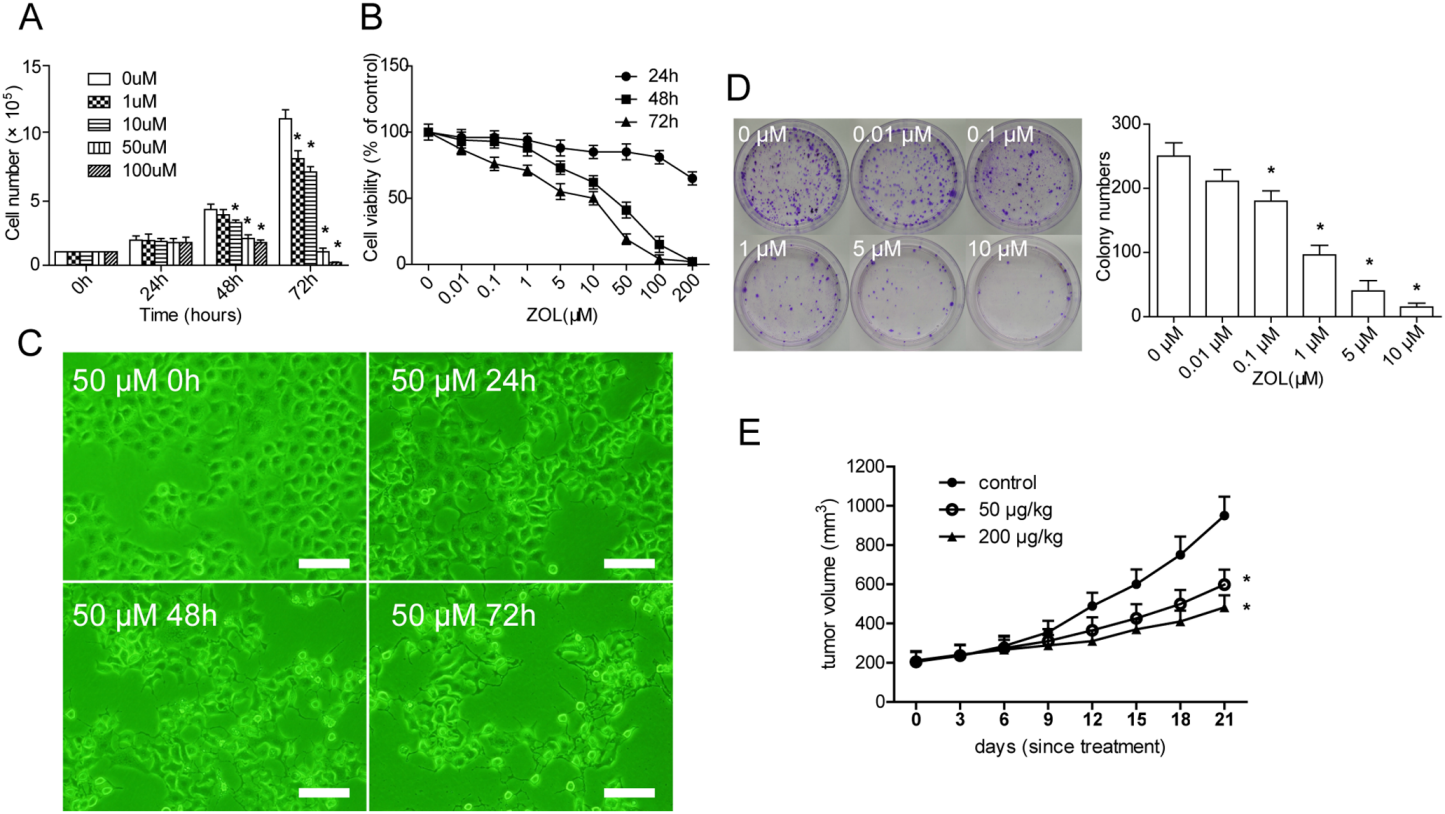
Effects of zoledronic acid (ZOL) on SACC-83 in vitro and in vivo. **A** After ZOL treatment, the total number of live SACC-83 cells was markedly decreased both dose and time dependently. **B** Cell viability assessed by Cell Counting Kit-8 assay after treatment with ZOL. **C** Representative phase-contrast images of SACC-83 cells treated with 50 µM ZOL at the indicated time points. Scale bar = 100 µm. **D** Colonies were stained with crystal violet, followed by scoring of colony numbers. **E** The growth curves of tumors in different group after the first treatment. ZOL inhibited the growth of SACC-83 xenograft tumor (n = 5 tumors). Error bars show the mean ± standard deviation. *Differences with *p*<0.05 are considered statistically significant.

Treatment with ZOL significantly reduced SACC-83 cell numbers compared with the control cells. Cell numbers in cultures treated with 50 or 100 µM ZOL decreased steadily over the ensuing 2 days, suggesting that cell death was occurring under these conditions (Fig. 1A). To further test the cytotoxic potential of ZOL, cells were incubated with a range of concentrations of ZOL followed by a CCK-8 test to determine their viability. ZOL treatment reduced cell viability both dose and time dependently, with 100 µM ZOL resulting in a more than 90% decrease in cells treated for 3 days compared with untreated controls (Fig. 1B). The treated cells also showed morphologic changes, with shrinkage and neurite extension (Fig. 1C).

Because of the significant cytotoxic effect of 50 and 100 µM ZOL on cell viability, we next tested whether lower concentrations of ZOL affected the colony forming ability of SACC-83 cells, with a colony forming assay using 0.01. 0.1, 1, 5 or 10 µM of ZOL in normal medium. After 14 days of culture, cells in the control group had formed a large number of colonies, whereas cells treated with 10 µM ZOL formed very few colonies (Fig. 1D). Also, the size of the colonies in the ZOL treatment groups was visibly reduced.

### Inhibition of xenograft tumor growth by ZOL

The in vivo antitumor activity of ZOL was examined in nude mice xenografted with SACC-83 cells. Consistent with the results from in vitro cell culture, the volumes of subcutaneous xenograft tumors in ZOL-treated groups were decreased to 62.9% (50 µg/kg group) and 50.7% (200 µg/kg group) of that of tumors in control group at the end of the 3-week treatment (Fig. 1E). We did not observe body weight loss in the treatment groups, indicating that the doses of ZOL used in the study were not toxic to the tested animals.

### Cell cycle arrest induced by ZOL

**Figure 2:**
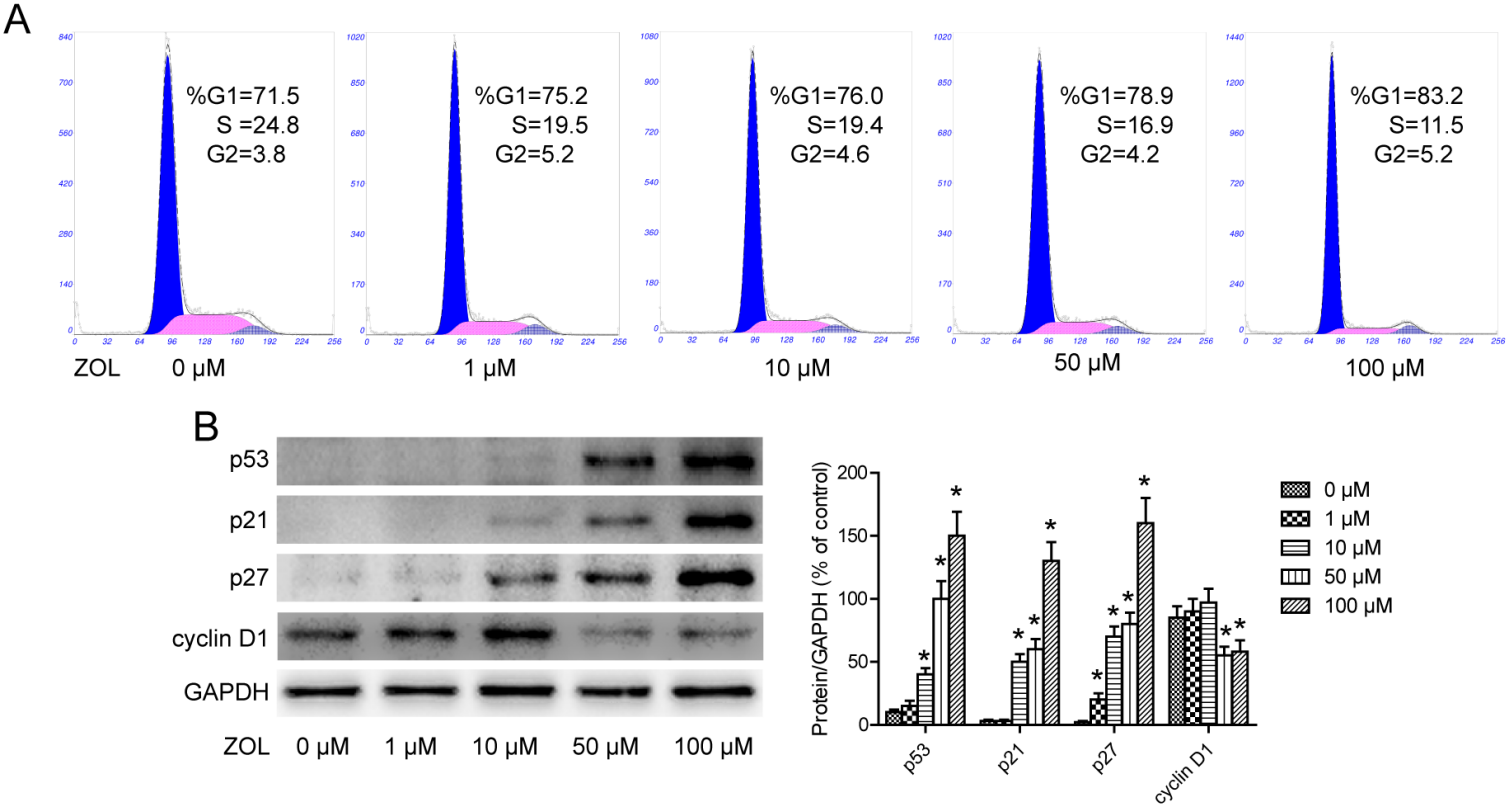
Cell cycle arrest induced by zoledronic acid (ZOL). **A** After 24**B** Effect of ZOL on cell cycle regulatory genes. SACC-83 cells were treated with ZOL (0–100 µM) for 24 h. Expression of cyclin D1, p21^CIP1^, p27^KIP1^ and p53 was measured by western blotting.

To explore the mechanisms of the cytotoxic effects of ZOL, drug-induced changes in cell cycle distribution were examined. We subjected ZOL treated cells to cell cycle analysis by PI staining and flow cytometry. Compared with the cell cycle profiles of untreated cells, ZOL induced an increase of cells in G0/G1 phase from 71.5% to 83.2%, indicating G1 cell cycle arrest (Fig. 2A). In agreement with this, we observed a decrease of cells in S+G2/M phase, from 28.6% to 16.7%. Statistically significant decreases in proliferation index, PI = [(S+G2M)/(G0/G1+S+G2M)]×100%, and S-phase fraction index, SPF = [S/(G0/G1+S+G2M)]×100%, were found in the each ZOL-treated group compared with untreated group (p<0.05). These findings indicate that ZOL may inhibit proliferation of SACC-83 cells by modulating cell cycle regulators.

To investigate the molecular mechanisms involved in the cell cycle block, western blot analysis was performed. As shown in Figure 2B, a G0/G1 block of the cell cycle in ZOL treated cells was supported by marked downregulation of cyclin D1 and upregulation of p21, p27and p53, according to drug concentration.

### Apoptosis assays

**Figure 3:**
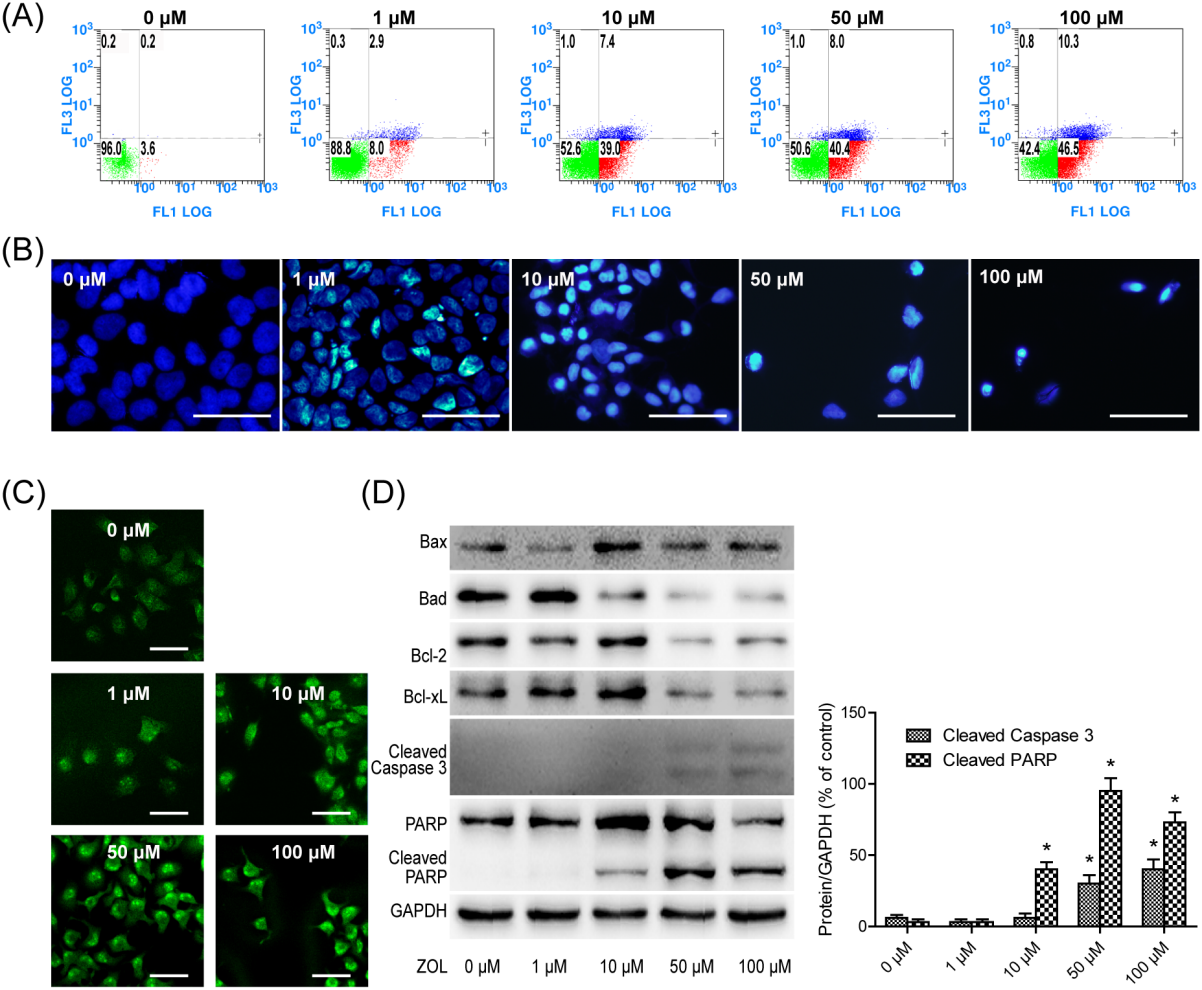
Zoledronic acid induces apoptosis after 48 h of treatment. **A** Annexin-V/propidium iodide double staining was performed to determine the apoptosis rate. Data are representative a result from three independent experiments. **B,** **C** 4,6-Diamidino-2-phenylindole dihydrochloride stained (Scale bar, 100 µm) and terminal transferase deoxyuridine triphosphate nick end labeled (Scale bar, 50 µm) cells were observed under a fluorescence microscope. **D** Expression of apoptosis related proteins was detected by western blotting.

To determine whether ZOL induced cell death by apoptosis, Annexin-V/PI, DAPI and TUNEL assays were employed. Annexin-V/PI staining was used to examine loss of integrity of cell membranes and the externalization of phosphatidylserine (PS), which are typical characteristics of apoptosis. ZOL induced a marked increase in the percentage of cells exhibiting externalized PS and loss of membrane integrity from 3.6% (control) to 46.5% (100 µM). When treated with ZOL, early or late apoptosis in SACC-83 cells was significantly increased compared with cells not treated with ZOL (*p*<0.05) (Fig. 3A). DAPI binds double-stranded DNA, producing blue fluorescence when viewed under ultraviolet light. Apoptotic cells are indicated by small, condensed nuclei. TUNEL staining is commonly used to detect DNA fragmentation, which is a hallmark of apoptosis. The numbers of DAPI/TUNEL positive cells were markedly increased after 48 h of treatment, further supporting the notion that ZOL treatment induced dose dependent apoptosis (Fig. 3B, C).

To evaluate the expression of apoptosis related proteins, cell lysates were analyzed by western blotting (Fig. 3D). Expression of Bcl-xL and Bcl-2 was significantly decreased in ZOL treated SACC-83 cells. By contrast, expression of Bax was significantly increased with this treatment. Expression of Bad was increased with a low dose of ZOL (1 µM) and decreased with high doses. Next, we determined the activated form of caspase-3 and its intracellular substrate PARP, both of which are considered biochemical markers of apoptosis. Increased cleavage of caspase-3 and degradation of PARP by caspase-3 into 89 kDa fragments were observed after 48 h of incubation (Fig. 1E).

### ROS and autophagy associated with ZOL-induced apoptosis of SACC-83

**Figure 4:**
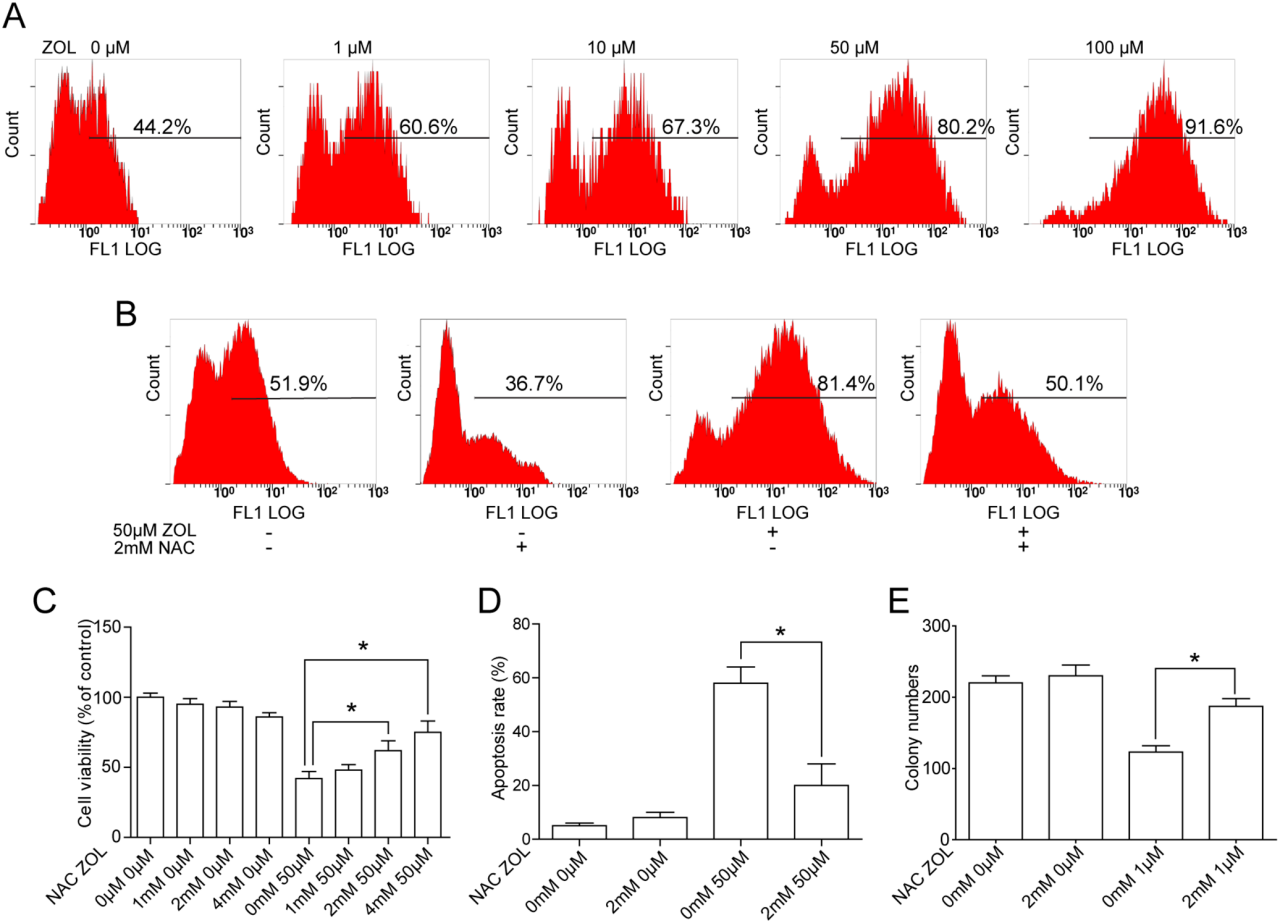
Zoledronic acid (ZOL) increased reactive oxygen species (ROS) production in SACC-83 cells. **A** ROS levels were measured by flow cytometry after incubation with dichlorodihydrofluorescein diacetate (DCFH-DA) fluorescent probe. **B** N-acetylcysteine (NAC) eliminated ZOL-induced ROS generation. Cells were protected by NAC (2 mM) with 12 h pretreatment when co-incubated with ZOL for another 48 h. ROS levels were determined by DCFH-DA staining. **C,** **D** Cells were pretreated with NAC for 12 h, then co-treated with or without 50 µM ZOL for another 48 h. The impact of ZOL and NAC on cell viability and apoptosis were determined by Cell Counting Kit-8 assay and 4,6-diamidino-2-phenylindole dihydrochloride staining, respectively. **E** Cells were pretreated with NAC for 12 h, then co-treated with or without ZOL for 14 d. The number of colonies formed was measured. All experiments were performed independently in triplicate per experimental point; representative results are shown. *Differences with *p*<0.05 are considered statistically significant.

We used the fluorescent probe DCFH-DA to measure the generation of intracellular ROS in ZOL treated SACC-83 cells. As shown in Figure 4A, when cells were treated with ZOL, DCF derived fluorescence was observed to increase from 44.2% (control) to 91.6% (100 µM). Then, we tested the effect of the ROS scavenger NAC on ZOL treated SACC-83 cells. NAC suppressed the generation of ROS induced by ZOL and partially blocked the ZOL-induced decrease of cell viability, increase of apoptosis and suppression of colony formation (Fig. 4B, C, D, E).

**Figure 5:**
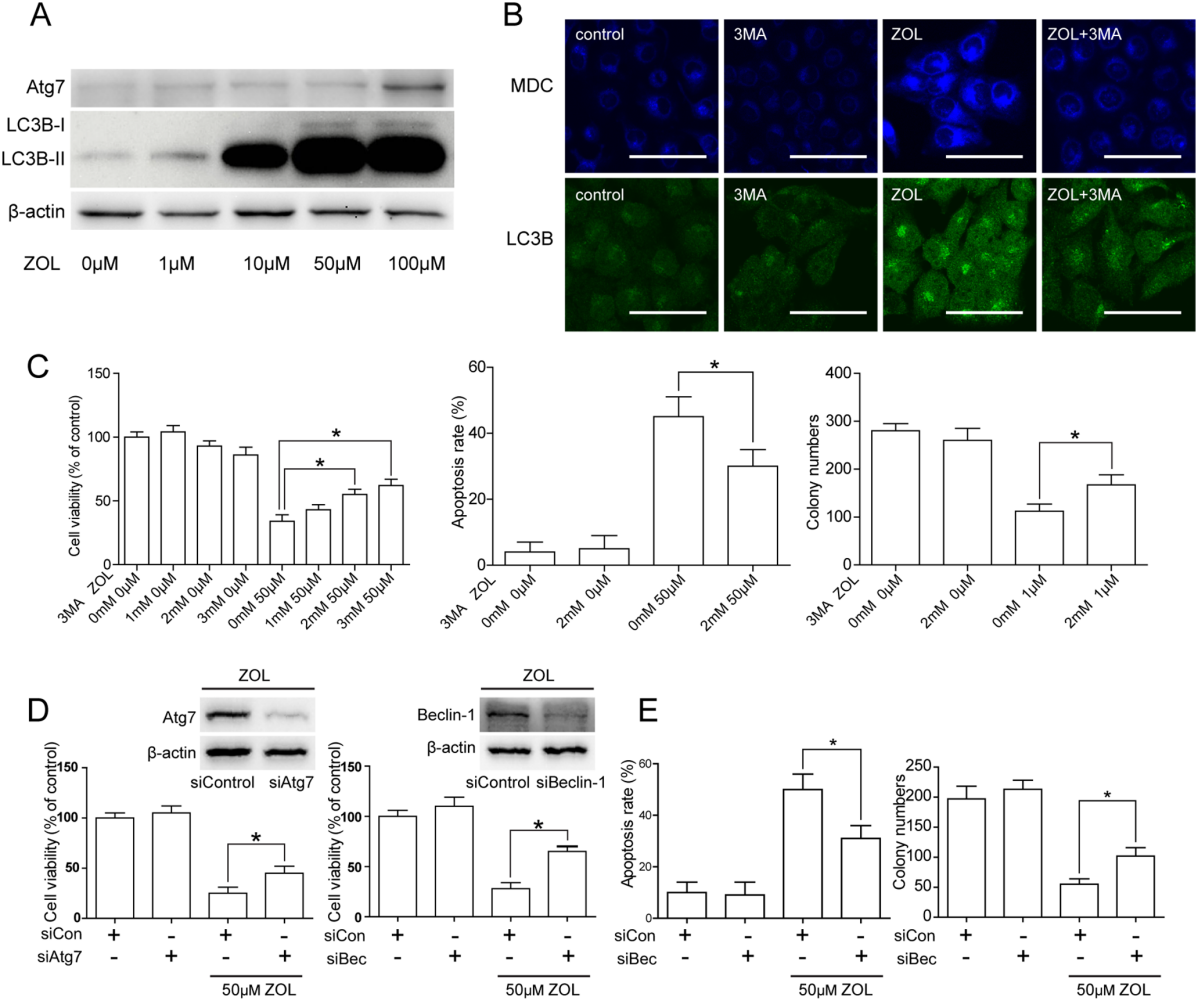
Inhibition of zoledronic acid (ZOL)-induced autophagy partially reversed SACC-83 apoptosis and inhibition of colony formation. A SACC-83 cells were treated with various doses of ZOL for 48 h and then LC-3B and Atg7 protein were determined by western blotting. B Cells were pretreated with 5 mM 3-methyladenine (3-MA) for 12 h before exposure to 50 µM ZOL for 48 h, then immunofluorescent staining for monodansyl cadaverine (MDC) and LC-3B was observed under a confocal microscope. Scale bar = 50 µm. C Impact of 5 µM 3-MA on the viability, apoptosis and colony formation of ZOL treated cells was measured by Cell Counting Kit-8 (CCK-8) assay, 4,6-diamidino-2-phenylindole dihydrochloride staining and a colony formation assay, respectively. D Cells were transfected with Atg7 or Beclin-1 small interfering RNA (siRNA) for 24 h before exposure to 50 mM ZOL for 48 h. Cell viability was measured by CCK-8 assay. E Effect of Beclin-1 siRNA on apoptotic cell death and colony formation. All data are representative of at least three independent experiments. *Differences with *p*<0.05 are considered statistically significant.

The autophagy specific markers LC-3B and Atg 7 were used to examine levels of autophagy by western blot analysis. As shown in Figure 5A, upregulation of Atg 7 and LC-3B demonstrated increased formation of autophagosomes in a dose dependent manner in SACC-83 cells. We used MDC staining and LC-3B immunofluorescence to determine whether autophagy was involved. Treatment with ZOL augmented the intensity of the immunofluorescence on MDC and LC-3B staining, which was partially reversed by the autophagy antagonist 3-MA (Fig. 5B). 3-MA also reduced apoptosis, and increased cell viability and colony numbers (Fig. 5C).

The role of autophagy in ZOL mediated cytotoxicity was further studied by knocking down Atg7 or Beclin-1 expression using siRNAs. As shown in Figure 5D, expression of Atg7/Beclin-1 was markedly suppressed in SACC-83 cells transfected with Atg7/Beclin-1 siRNA compared with those treated with control siRNA. In addition, the cytotoxic effect of ZOL was significantly decreased by blocking Atg7/Beclin-1 expression (Fig. 5D). Similarly, apoptosis and inhibition of colony formation induced by ZOL were reversed significantly following knock down of Beclin-1 protein (Fig. 5E).

### ZOL synergizes with paclitaxel/cisplatin to enhance cytotoxicity

**Figure 6:**
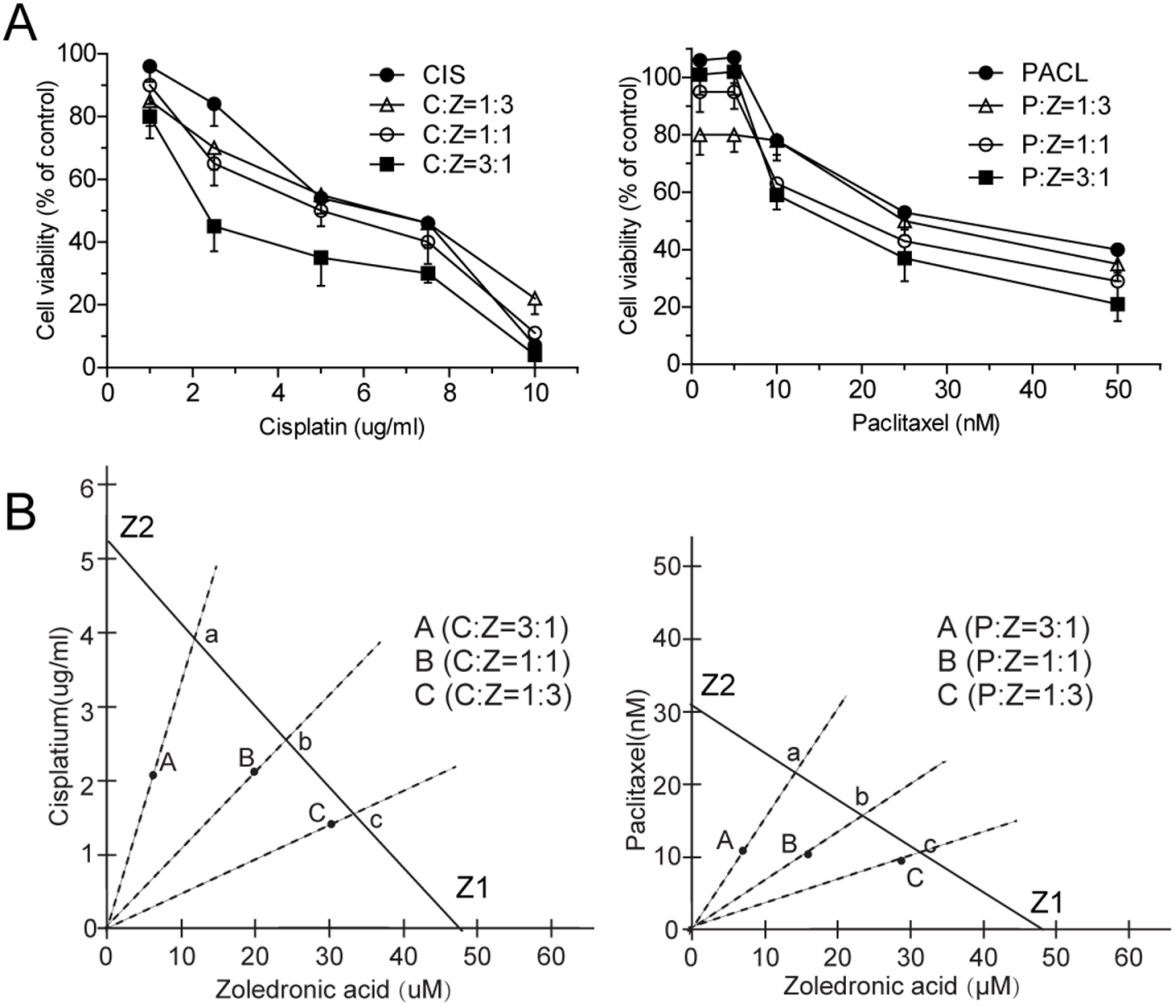
Zoledronic acid (ZOL) enhances cisplatin/paclitaxel-induced cytotoxicity in SACC-83 cells. Cells were co-treated with ZOL and cisplatin/paclitaxel for 48 h. **A** Cell viability was examined by Cell Counting Kit-8 assay. **B** Isobolographic analysis of the cytotoxic effects of ZOL and cisplatin/paclitaxel on SACC-83 cells.

Using a CCK-8 assay, we obtained IC50s for ZOL (47.66 µM), paclitaxel (31.50 µM) and cisplatin (5.20 µg/ml). We then treated cells with 3∶1, 1∶1 and 1∶3 ratios of ZOL and paclitaxel/cisplatin according to IC50. A dose–response relationship for the mixtures was obtained (Figure 6A) and the Zmix values for ZOL and cisplatin at 1∶3, 1∶1 and 3∶1 were 2.10, 2.13 and 1.22 µg/ml cisplatin, respectively; the calculated theoretical additive IC50 yielded Zadd values for ZOL and cisplatin of 3.96, 2.62 and 1.33 µg/ml cisplatin, respectively. Zmix values for ZOL and paclitaxel at 1∶3, 1∶1 and 3∶1 were 10.84, 10.67 and 8.36 µM paclitaxel, respectively; the calculated theoretical additive IC50 yielded Zadd values for ZOL and paclitaxel of 23.62, 15.61 and 11.17 µM paclitaxel, respectively.

Zmix values for the two investigated ZOL:paclitaxel/cisplatin ratios are shown in Figure 6B. Here, A, B and C represent the Zmix when the ZOL:paclitaxel/cisplatin ratio was 1∶3, 1∶1 or 3∶1, respectively. A, B and C lie below the straight line between Z1 and Z2, indicating that the combination of ZOL and paclitaxel/cisplatin at these three ratios has a synergistic effect on cell viability (a, b and c indicate the theoretical additive point at the different ratios). When we compared relative median potency between the mixtures and the theoretical additive combinations, we found that the ZOL:paclitaxel/cisplatin ratio of 1∶3 was statistically significantly greater. This means that this mixture had a statistically significant synergistic effect on cell viability.

## Discussion

ZOL has been widely used to treat osteoporosis [29], [30] and bone metastasis [31]. Recent studies show that ZOL also has direct antineoplastic activity in many human tumors [19], [32]–[36]. In the present study, we report the first experimental evidence of cytotoxicity of ZOL on the SACC-83 cell line. ZOL concentrations of 50 and 100 µM strongly reduced tumor cell viability after 3 days of treatment. This dramatic reduction could be caused by slowing of cell proliferation as well as by induction of apoptosis. We next observed the effect of ZOL on cell cycle distribution and apoptosis in SACC-83 cells. Cells cycle analysis of ZOL treated cells that had not yet detached from the cell culture surface demonstrated G0/G1 cell cycle arrest, which suggests a disturbance of cell cycle progression and decreased cell proliferation. The G0/G1 cell cycle arrest caused by ZOL in SACC-83 cells is consistent with findings in ZOL treated HCT-116 colon carcinoma cells and A549 non-small cell lung cancer cells [19], [37]. However, the effect differs from that observed in ZOL treated hepatocellular carcinoma cells, normal human oral keratinocytes and cholangiocarcinoma cells [32], [38], [39], in which the arrest occurred in S phase. Because ZOL has been reported to reduce cell viability with and without induction of apoptosis [16], [19], [39]–[43], we investigated whether apoptosis occurred in ZOL treated SACC-83 cells. The increased in cleaved Caspase-3 and PARP protein demonstrated apoptosis in a dose dependent manner. Bcl-2 family protein is important for its role in apoptosis, and the interplay between anti-apoptosis and pro-apoptosis proteins is vital for the process. As shown in Figure 3D, Bax was upregulated whereas Bcl-2 and Bcl-XL were downregulated in ZOL treated cells. The pro-apoptosis protein Bad was upregulated at a low dose of ZOL (1 µM) and decreased at higher doses (10, 50 and 100 µM). The detailed function of Bad or p-Bad protein in ZOL-induced apoptosis may need to be further explored.

Increased ROS generation has been associated with carcinogenesis in solid tumors, and excessive ROS levels can trigger cell death. Production of ROS is an underlying mechanism of chemotherapy and radiotherapy [44]. Previous studies have shown that combined application of ZOL with panobinostat or ionizing radiation significantly enhanced apoptosis by elevating ROS levels [45], [46]. Here, we found that ZOL alone can induce apoptotic cell death and inhibit colony formation accompanied by an increase of ROS. Apoptosis and inhibition of colony formation were decreased by the ROS scavenger NAC. These findings suggest that ROS are involved in apoptosis and inhibition of colony formation in ZOL treated SACC-83 cells. Autophagy can have a pro-survival or pro-death role depending on the tumor cell or development stage. ZOL has been shown to induce autophagic cell death in human prostate cancer cells [36]; using the MTT method, the study showed that cell growth was rescued by an apoptosis or autophagy inhibitor in ZOL treated cells, but the relationship between autophagy and apoptosis was not explored. In the present study, ZOL induced apoptosis and inhibited colony formation accompanied by increased autophagy. Apoptosis and inhibition of colony formation were reduced by the administration of an autophagy antagonist during ZOL treatment, indicating that autophagy played a pro-death role by facilitating apoptosis.

Isobolographic analysis is a valuable method for analyzing synergistic effects in combined treatment. It has been used to identify the interaction between two drugs regardless of the mechanisms of action of the individual drugs [47]–[49]. Identification of a synergistic interaction allows the use of lower doses of the constituents of the combination, which may reduce adverse reactions [28]. ZOL has been reported to have synergistic effects with paclitaxel/cisplatin [49]–[52]. In the present study, the dose–response curve and isobologram analysis indicated evidence of a synergistic effect between ZOL and paclitaxel/cisplatin on SACC-83 cell viability. Combined use of a low proportion of ZOL with a high proportion of paclitaxel/cisplatin had greater cytotoxicity in SACC-83 cells.

## Conclusion

Our data indicate that ZOL inhibited SACC-83 xenograft tumor growth in nude mice. In vitro, ZOL induced apoptosis and reduced colony formation in SACC-83, the process in which ROS and autophagy were involved. In addition, ZOL was shown to synergize with paclitaxel/cisplatin in reducing the viability of SACC-83 cells. These results suggest that ZOL may be beneficial in the treatment of patients with SACC.
